# Construction of Cardiac Tissue Rings Using a Magnetic Tissue Fabrication Technique

**DOI:** 10.3390/ijms11082910

**Published:** 2010-08-10

**Authors:** Hirokazu Akiyama, Akira Ito, Masanori Sato, Yoshinori Kawabe, Masamichi Kamihira

**Affiliations:** Department of Chemical Engineering, Faculty of Engineering, Kyushu University, 744 Motooka, Nishi-ku, Fukuoka 819-0395, Japan; E-Mails: hakiyama@chem-eng.kyushu-u.ac.jp (H.A.); akira@chem-eng.kyushu-u.ac.jp (A.I.); 2TE10138G@s.kyushu-u.ac.jp (M.S.); kawabe@chem-eng.kyushu-u.ac.jp (Y.K.)

**Keywords:** cardiac tissue engineering, cardiomyocytes, magnetic nanoparticle, magnetic force

## Abstract

Here we applied a magnetic force-based tissue engineering technique to cardiac tissue fabrication. A mixture of extracellular matrix precursor and cardiomyocytes labeled with magnetic nanoparticles was added into a well containing a central polycarbonate cylinder. With the use of a magnet, the cells were attracted to the bottom of the well and allowed to form a cell layer. During cultivation, the cell layer shrank towards the cylinder, leading to the formation of a ring-shaped tissue that possessed a multilayered cell structure and contractile properties. These results indicate that magnetic tissue fabrication is a promising approach for cardiac tissue engineering.

## 1. Introduction

It is well known that the human heart has a limited capacity for regeneration after myocardial infarction. Although heart transplantation is a therapeutic option, it is restricted due to a lack of suitable donor organs. Even in the case of a successful transplantation, the resultant lifelong immune suppression causes a number of serious side effects. Therefore, myocardium tissues artificially reconstructed using tissue engineering techniques provide a new muscle material for tissue replacement therapy [1,2]. Such artificial tissue constructs can also serve as important tools for *in vitro* cardiac tissue models to study cardiac developmental biology and to screen pharmacological agents for heart disease treatments. In addition to applications in the biomedical field, various bio-actuators, in which kinetic forces generated by engineered cardiac tissues are utilized as driving forces, have been developed [3–5]. Thus, significant efforts are currently focused on developing three-dimensional (3D) cardiac tissue models. The most common approach is an extracellular matrix (ECM)-based procedure in which spontaneous 3D tissue formation can be induced from a mixture of cardiomyocytes and ECM precursors such as collagen and Matrigel [6–8]. ECM components play essential roles in the development and signaling of cardiac tissues and also contribute to the enhancement of mechanical strengths with maintenance of tissue flexibility [9]. Indeed, cardiac tissues fabricated by this method exhibited a spontaneous rhythmic contractility, which was additionally improved by an application of cyclic stretch, leading to cardiac differentiation [7]. However, in the engineered cardiac tissues, cardiomyocytes were mainly distributed at the tissue periphery and were less compact than native myocardium, which can limit the further development of cardiac functionality. Thus, technological advances are necessary in order to establish a 3D cardiac model that is structurally analogous to native tissues in terms of cell distribution and density.

Magnetite cationic liposomes (MCLs) were previously developed by encapsulating 10 nm magnetite nanoparticles into cationic liposomes, which enhanced the binding ability to negativelycharged cell membranes [10]. For the application of MCLs in tissue engineering, we have proposed a “magnetic force-based tissue engineering (Mag-TE)” technique [11], in which the MCL-labeled cells are magnetically assembled to form a 3D tissue-like structure. The major advantage of the Mag-TE technique is the induction of cell-dense tissues mimicking native tissues, as demonstrated in the fabrication of cardiomyocyte [12] and myoblast cell sheets [13]. Accordingly, we speculated that a combination of the Mag-TE technique and an ECM-based procedure would overcome limitations of the conventional ECM-based procedure. Regarding muscle tissue rings fabricated by this combination approach [14], myoblast cells were densely organized, and ECMs enhanced mechanical strength sufficiently to support physiological loads and to promote skeletal muscle differentiation.

The aim of this study was to investigate the combination approach utilizing the Mag-TE technique and an ECM-based procedure in the application of cardiac tissue engineering and to evaluate the structural and contractile properties of the resultant engineered cardiac tissues.

## 2. Experimental Section

### 2.1. Primary Culture of Neonatal Rat Cardiomyocytes

Primary neonatal rat cardiomyocytes were isolated using a neonatal cardiomyocyte isolation kit (Worthington Biochemical, Lakewood, NJ, USA) according to the published procedure [12]. Briefly, ventricles from 2–4 day-old Sprague-Dawley rats (Japan SLC, Inc., Hamamatsu, Japan) were incubated for 16–20 h at 4 °C in Hank’s balanced salt solution containing trypsin (50–100 μg/mL), followed by digestion with collagenase (75 U/mL) for 40 min at 37 °C. Isolated cells were suspended in Medium 199 (Invitrogen, Carlsbad, CA, USA) containing 10% heat-inactivated fetal bovine serum (FBS; Biowest, Nuaille, France), 0.1 mg/mL streptomycin sulfate and 100 U/mL penicillin G potassium (Wako Pure Chemical Industries, Osaka, Japan), and plated to allow preferential attachment of non-cardiomyocytes. After 1 h incubation, non-adherent cells were collected and used for subsequent experiments.

All animal experiments were approved by the Ethics Committee for Animal Experiments of the Faculty of Engineering, Kyushu University (A19-114-1).

### 2.2. Preparation of MCLs

Magnetite (Fe_3_O_4_; average particle size, 10 nm) used as the core of MCLs was obtained from Toda Kogyo (Hiroshima, Japan). Cationic liposomes of MCLs were composed of *N*-(α-trimethylammonioacetyl)-didodecyl-D-glutamate chloride (TMAG), dilauroylphosphatidylcholine (DLPC) and dioleoylphosphatidyl-ethanolamine (DOPE) in a molecular ratio of 1:2:2. MCLs were prepared as described previously [10]. The average diameter of MCLs was 150 nm [12].

### 2.3. MCL-Labeling of Cardiomyocytes

Isolated cardiomyocytes were suspended in medium containing the MCLs (0, 25, 50, 100 and 200 μg/mL) at a cell density of 1 × 10^6^ cells/mL and incubated for 1 h at 4 °C. To determine the amount of MCLs captured by the cells, MCL-labeled cells were collected by centrifugation, and the iron concentration within the cell pellets was measured using the potassium thiocyanate method [15]. To investigate the effect of MCL-labeling on cell viability, cardiomyocytes with or without MCLlabeling were seeded into wells of a 96-well plate at a cell density of 1.5 × 10^4^ cells/well. After 4-day culture, cell metabolic activity was measured using a commercially available kit based on the WST-8 assay (Cell Counting Kit 8, Dojindo Laboratories, Kumamoto, Japan) according to the manufacturer’s protocol and used as the index for cell viability. The absorbance of each well at 490 nm was measured using a microplate reader (Model550, Bio-Rad Laboratories, Hercules, CA, USA). The absorbance of control wells containing cells without MCL-labeling was also measured and defined as 100% cell viability. Viability of MCL-labeled cells was determined as the relative value to the absorbance of the control wells.

### 2.4. Fabrication of Cardiac Tissue Rings by Combining the Mag-TE and ECM-Based Techniques

**Figure 1:**
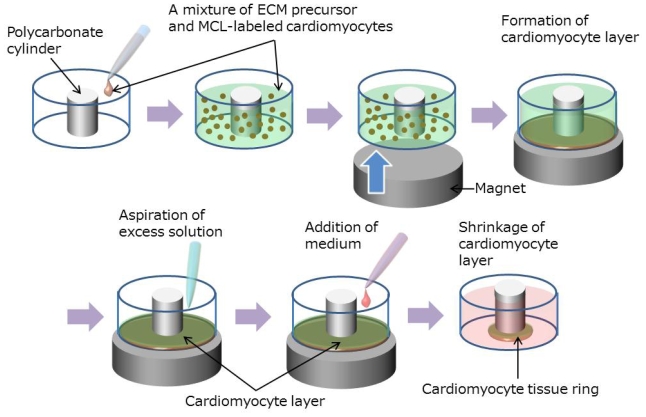
A procedure for fabrication of a cardiac tissue ring by combining Mag-TE and ECM-based techniques. A mixture of diluted ECM precursor and MCL-labeled cardiomyocytes was cast into a well of a 24-well ultra-low attachment plate containing a polycarbonate cylinder fixed in the center of each well. Immediately thereafter, a magnet was placed underneath the wells to attract the MCL-labeled cardiomyocytes to the culture bottom, enabling the removal of excess amounts of ECM precursor from the upper side of the formed cell layer. The remaining ECM within the cell layer was then hardened, and the medium was added. During the culture, the cell layer gradually shrank towards the cylinder, resulting in the formation of ring-shaped cardiac tissue.

A procedure for fabrication of cardiac tissue rings by combining the Mag-TE and ECM-based techniques is illustrated in Figure 1. Collagen solution was prepared by mixing type I collagen (Nitta Gelatin, Osaka, Japan), 10 × Medium 199, neutralization buffer (0.05N NaOH) and FBS in a volume ratio of 7:1:1:1. An MCL-labeled cell suspension (2 × 10^6^ cells in 100 μL) was mixed with an ECM precursor solution composed of 170 μL collagen solution and 30 μL Matrigel basement matrix (BD Biosciences, Franklin Lakes, NJ, USA). The final concentration of type I collagen was adjusted to 0.5 mg/mL. Subsequently, the mixture (300 μL) was cast into a well of a 24-well ultra-low attachment plate, in which polycarbonate cylinders (diameter, 8 mm; height, 5 mm) were fixed at the center of each well. Immediately thereafter, a magnet was placed underneath the wells to accumulate MCL-labeled cardiomyocytes onto the culture bottom. After the formation of cell layers, excess ECM precursor on top of the cell layer was carefully aspirated using a micropipette. The procedure was conducted under cold conditions. The remaining ECM within the cell layer was then hardened by incubation for 1 h at 37 °C. Thereafter, medium was added to each well. After 3–5 days culture, the cell layer completely shrank around the cylinder, resulting in the formation of a ring-shaped tissue. The tissue ring was removed from the cylinder and hooked around two stainless steel pins (0.6 mm diameter; Shiga, Tokyo, Japan) that were positioned 12 mm apart from each other, and cultivation was further continued. The medium was changed every day.

After seven days of culture, the cardiac tissue rings were washed three times with phosphatebuffered saline (PBS) and fixed in 4% paraformaldehyde (PFA). For scanning electron microscopy (SEM), the tissue was imaged using a microscope (VE-9800; Keyence, Osaka, Japan). For histological analysis, the cardiac rings were embedded in paraffin. Thin sections (4 μm) were stained with hematoxylin and eosin (H&E) [13].

### 2.5. Measurement of Contractile Force

After 7-day culture, the cardiac tissue ring was hooked around two stainless steel pins (0.3 mm diameter; Shiga). Here, one of the pins was attached to a force transducer (AE-801; SonorOne, Sausalito, CA, USA), and the other was connected to a micro-manipulator. During testing, the medium was maintained at 30–32 °C using a heated aluminum platform (HI-1000; AsOne, Osaka, Japan). Electric pulses were controlled by a personal computer with specially designed LabView software (National instruments, Austin, TX, USA), and the applied electric pulse and measured force were recorded to the same computer. The tissues were electrically stimulated at a voltage of 1 V/mm with a pulse duration of 10 ms. For the investigation of the effect of tissue stretch on contractile force, the tissues were strained from 0 to 20% of original length (12 mm). To determine the effect of calcium concentration on contractile force, media containing various calcium concentrations (0–15 mM) were prepared from calcium-free medium containing 5 mM *O*,*O*′-bis(2-aminoethyl)ethyleneglycol- *N*,*N*,*N*′,*N*′-tetraacetic acid (EGTA; Dojindo Laboratories).

### 2.6. Statistical Analysis

All data are expressed as means ± SD. Statistical comparisons were evaluated using one-way analysis of variance (ANOVA). A difference was considered to be significant when the calculated *P* value was less than 0.05 as indicated by asterisks.

## 3. Results and Discussion

### 3.1. MCL-Labeling of Cardiomyocytes

**Figure 2:**
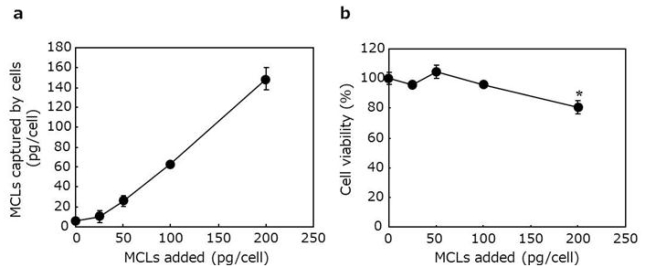
MCL-labeling of cardiomyocytes. (**a**) Measurement of MCL amount captured by the cells. Isolated cardiomyocytes were suspended in the medium containing various MCL concentrations (0, 25, 50, 100 and 200 pg/cell), and incubated for 1 h at 4 °C. Subsequently, the magnetite amount captured by the cells was measured using the potassium thiocyanate method. (**b**) The effect of MCL-labeling on cell viability. Cardiomyocytes with or without MCL-labeling were seeded into wells of a 96-well plate. After 4-day culture, cell viability was measured using the WST-8 assay. Data are expressed as mean ± SD (n = 3). * *P* < 0.05 *vs.* the group without MCLs.

In our previous study, in order to magnetically label cells, MCLs were added to cells that had adhered onto the culture surfaces. In the present study, cardiomyocytes were labeled with MCLs by incubating the cells with MCLs in suspension to avoid cell damage at the cell harvest using a digestive enzyme (*i.e.*, trypsin). As shown in Figure 2a, the amount of MCLs captured by the cells increased linearly when added in the range of 25–200 pg/cell. When MCLs were added at 100 pg/cell to a suspension of cardiomyocytes, the amount of MCLs captured by the cells was 62.9 pg/cell, which was much higher than that captured by cardiomyocytes that had adhered on the culture surface (18 pg/cell) [12]. The enhancement of MCL uptake was attributable to frequent contact of MCLs with cells in suspension culture. Thus, the present method was effective for MCL-labeling of cardiomyocytes. On the other hand, a significant decrease in cell viability was observed at an MCL concentration of 200 pg/cell (Figure 2b). Accordingly, in the following experiments, MCLs were added at 100 pg/cell for magnetic labeling of cardiomyocytes.

### 3.2. Fabrication of Cardiac Tissue Rings by Combining Mag-TE and ECM-Based Techniques

**Figure 3:**
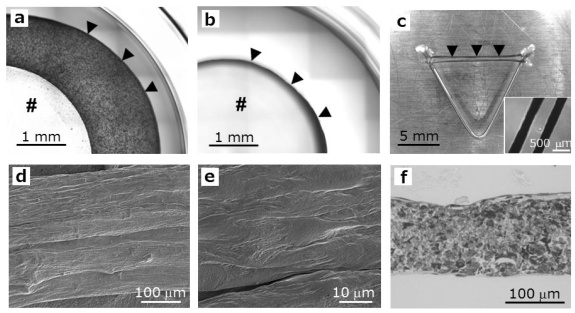
Cardiac tissue rings fabricated by combining Mag-TE and ECM-based procedures. (**a**, **b**) Bright-field micrographs of cell layers after 1-day (a) and 3-day fabrications (b). The layer gradually shrank towards the cylinder (#), resulting in the formation of cardiac tissue rings (arrowheads). (**c**) Macroscopic photograph of a cardiac tissue ring cultured around stainless steel pins. Arrowheads indicate a cardiac tissue ring. The inset shows a bright-field micrograph of the cardiac tissue. (**d**, **e**) Structural images of cardiac tissue rings. The tissue was observed using SEM at low- (d) and high-power magnification (e). (**f**) Bright-field micrograph of an H&E-stained section of a cardiac tissue ring.

Because tissues that are designed to have a cylindrical geometry facilitate the evaluation of muscle tissues, ring-shaped cardiac tissues were constructed by combining Mag-TE and ECM-based techniques according to a procedure illustrated in Figure 1. A mixture of diluted ECM precursor and MCL-labeled cardiomyocytes was pipetted into wells of a 24-well ultra-low attachment plate containing polycarbonate cylinders fixed in the center of each well. The cells were then attracted to the bottom of each well, allowing formation of a cell layer (Figure 3a). During the culture, the layers gradually shrank towards the polycarbonate cylinder (Figure 3b), leading to the formation of ring-shaped tissues. The shrinkage of cell layer, caused by traction of cardiomyocytes, was also observed in collagen gel-based cardiac tissue engineering [6,7]. The rings were then removed and hooked around stainless steel pins in order to supply oxygen and nutrients (Figure 3c). After transfer to the pins, the cardiac tissues exhibited spontaneous contraction, which was sometimes macroscopically observable when the tissues were released from the pins on day 7 after construction. The images of cardiac tissue rings by SEM are shown in Figures 3d and e. Due to the tension generated within the tissue, the longitudinal alignment of the cardiomyocytes was observed at the outer surface of the ring tissue. This was consistent with previous reports [6–8], in which the conventional ECM-based procedures were employed for cardiac tissue fabrication. The H&E-stained section of a cardiac tissue ring after 7-day cultivation is shown in Figure 3f. An earlier report showed that a tissue thickness below 128 μm from the tissue surface exposed to medium was necessary for cardiomyocytes to remain viable [16]. The average thickness of cardiac tissue rings was 247 ± 32 μm (n = 3), and there were no substantial necrotic areas within the tissues (Figure 3f). When the cells were accumulated by the Mag-TE procedure, the cardiomyocytes were densely packed within the ring tissues containing ECM, indicating that the combination approach could mimic myocardium in terms of cell density and distribution as compared with that prepared by the conventional ECM-based procedure. Additionally, immunohistological analysis revealed the localization of connexin 43, a major gap junction-related protein of cardiac muscle, at the membrane of cardiomyocytes within the ring tissues (data not shown), indicating that cell-cell communications were well-established.

The dynamic strength of tissues is an important consideration for a wide-range of applications. In our preliminary experiment, the cardiac tissue fabricated by the Mag-TE technique without ECM addition was easily broken by minute physical damage, which may be a major issue of scaffold-free tissue engineering approaches [17–19]. In our previous study, without coating with ECMs, most tissues were broken by physiological loads [14]. In this study, cardiac tissue rings did not form without ECMs. On the other hand, the cardiac tissue rings fabricated by Mag-TE with ECMs had enough strength for manipulation using forceps. Moreover, almost all of the tissues maintained at least for seven days without breakage, indicating that the present method was effective to construct muscle tissues with sufficient strength for manipulation.

### 3.3. Analyses of Contractile Properties of the Cardiac Tissue Rings

**Figure 4:**
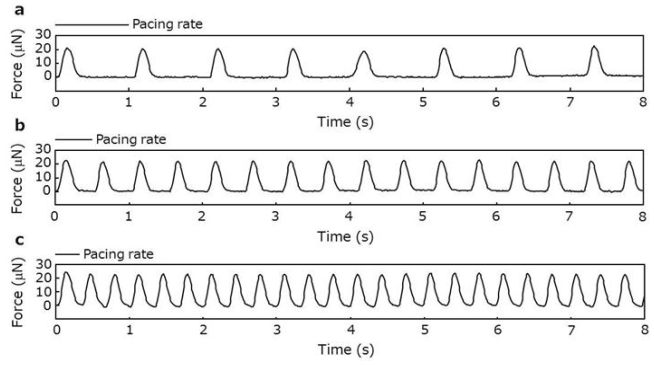
Contractile behavior of cardiac tissue rings. Electrical stimuli (10 ms, 1 V/mm) were applied at frequencies of 1 (**a**), 2 (**b**) and 3 Hz (**c**), and the contractile behaviors of cardiac tissue rings were recorded. Representative traces are shown.

**Figure 5:**
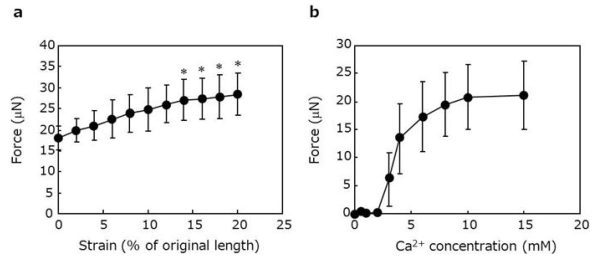
Functional analyses of cardiac tissue rings. (**a**) The effect of tissue stretch on contractile force. Cardiac tissue rings were strained from 0 to 20% of their original lengths (12 mm) using a micro-manipulator. Single pulses (10 ms, 1 V/mm) were applied at each point, and the contractile forces were recorded. Data are expressed as mean ± SD (n = 3). *P < 0.05 *vs.* the group without stretch. (**b**) The effect of Ca^2+^ concentration on contractile force. Media containing various Ca^2+^ concentrations were prepared from Ca^2+^-free medium containing 5 mM EGTA. Single pulses (10 ms, 1 V/mm) were applied in these media, and the contractile forces were recorded.

In order to assess the contractile properties of the cardiac tissue rings, the rings were removed from the incubator and hooked onto a device where one of the pins was attached to a force transducer, and the other was connected to a micro-manipulator. The tissues were able to generate contractile forces in response to electrical pulses (Figure 4). The forces increased gradually by the application of tissue stretch (Figure 5a). Significantly higher forces were achieved when tissue stretches above 14% strain of the original length were applied (14, 16, 18 and 20% strain caused 27.1 ± 4.9, 27.4 ± 5.0, 27.9 ± 5.1 and 28.5 ± 5.0 μN, respectively). Moreover, when strain was fixed at 20%, the forces decreased by increasing pulse frequency (data not shown), which is a coincident phenomenon with native cardiac tissues [20]. Furthermore, the force generation of the cardiac tissues was dependent on the extracellular Ca^2+^ concentration, as observed in heart muscle (Figure 5b). Taken together, the cardiac tissue rings fabricated by Mag-TE with ECMs exhibited contractile properties of myocardium.

For the application of cardiac tissues to versatile purposes, it is necessary to enhance contractile properties by mimicking the structure of natural cardiac muscle. In the present study, the functional cell-dense tissues were successfully constructed by the present procedure. However, the unidirectional cardiomyocyte alignment, which is important for muscular tissue to generate contractile forces, might be limited at the outer surface of the ring tissues. Zimmermann and co-workers demonstrated that cyclic stretching of cardiac tissues promoted longitudinal cell orientation, leading to the induction of tissues with greater contractile forces [7]. Thus, our next subject is to investigate the effect of cyclic stretching to the cardiac tissue rings on cardiomyocyte alignment and their contractile forces.

## 4. Conclusion

We developed a 3D cardiac tissue model by combining Mag-TE with ECM-based procedures. The cardiac tissue rings fabricated by this method exhibited several advantages: (1) the cardiomyocytes within the tissues were densely packed and distributed homogeneously, and partially showed unidirectional alignment; (2) the cardiac tissues had sufficient strength for manipulation, and (3) the cardiac tissues showed spontaneous contraction and were also electrically excitable. Thus, we believe that the cardiac tissue model established in this study may be a promising platform applicable for various purposes.
